# Artemether Combined with shRNA Interference of Vascular Cell Adhesion Molecule-1 Significantly Inhibited the Malignant Biological Behavior of Human Glioma Cells

**DOI:** 10.1371/journal.pone.0060834

**Published:** 2013-04-11

**Authors:** Ying-Bin Wang, Yi Hu, Zhen Li, Ping Wang, Yi-Xue Xue, Yi-Long Yao, Bo Yu, Yun-Hui Liu

**Affiliations:** 1 Department of Neurosurgery, Shengjing Hospital of China Medical University, Shenyang, Liaoning Province, People’s Republic of China; 2 Department of Neurobiology, College of Basic Medicine, China Medical University, Shenyang, Liaoning Province, People’s Republic of China; 3 Institute of Pathology and Pathophysiology, China Medical University, Shenyang, Liaoning Province, People’s Republic of China; UAE University, United Arab Emirates

## Abstract

Artemether is the derivative extracted from Chinese traditional herb and originally used for malaria. Artemether also has potential therapeutic effects against tumors. Vascular cell adhesion molecule-1 (VCAM-1) is an important cell surface adhesion molecule associated with malignancy of gliomas. In this work, we investigated the role and mechanism of artemether combined with shRNA interference of VCAM-1 (shRNA-VCAM-1) on the migration, invasion and apoptosis of glioma cells. U87 human glioma cells were treated with artemether at various concentrations and shRNA interfering technology was employed to silence the expression of VCAM-1. Cell viability, migration, invasiveness and apoptosis were assessed with MTT, wound healing, Transwell and Annexin V-FITC/PI staining. The expression of matrix metalloproteinase-2 (MMP-2), matrix metalloproteinase-9 (MMP-9) and phosphorylated Akt (p-Akt) was checked by Western blot assay. Results showed that artemether and shRNA-VCAM-1 not only significantly inhibited the migration, invasiveness and expression of MMP-2/9 and p-Akt, but also promoted the apoptosis of U87 cells. Combined treatment of both displayed the maximum inhibitory effects on the malignant biological behavior of glioma cells. Our work revealed the potential therapeutic effects of artemether and antiVCAM-1 in the treatments of gliomas.

## Introduction

Gliomas, the most common primary malignant tumors in the central nervous system, are refractory to the surgical therapy of extensive resection and are easy to be recurrent due to their highly invasive and infiltrative growth pattern [1]. The median survival after surgical resection alone is 6 months and only 7.5% of patients survived two years post-operatively [2]. Their biological characteristics are highly malignant, featuring strong proliferation, rapid migration, intensive invasion and a very poor prognosis. Radiotherapy and chemotherapy adjuvant to surgery have been standard in the treatments of gliomas [2]–[4]. Nevertheless, despite the significant advances in neuroimaging, neurosurgical techniques, radiotherapy and in the molecular understanding of tumorigenesis, the outcomes for patients with gliomas still remain unchanged for the last several decades [2], [4]. Gliomas, especially high grade gliomas display heterogeneity in genetic inherence and are often resistant to anti-tumoral drugs, resulting in the limitedness of chemotherapies [4], [5]. Therefore, it is absolutely important to develop new agents and therapies to prevent the proliferation, migration and invasion of gliomas. Under such background, the Chinese traditional medicine might bring new hope. Recently, agents extracted from Chines traditional medicine have been reported to have therapeutic effects against gliomas. For examples, Jian et al reported that panaxydol, isolated from the lipophilic fractions of Panax notoginseng, a well-known Chinese traditional medicine, inhibited the proliferation of C6 cells in a dose-dependent manner and induced p27 expression and differentiation in rat C6 glioma cells [6]. Shao et al reported that nanoparticles loaded with Curcumina, yellow pigment in the spice turmeric displayed pro-apoptosis effect against C6 cells [7].

In recent years, studies have demonstrated that artemisinin and its derivatives had a significant cytotoxic effects toward cancer cells and could reverse multiple drug resistance of tumors [8]–[10]. Artemisinin was first isolated from leaves of Artemisia annua, a Chinese traditional herb, by Chinese pharmacists in 1971 [11]. Artemether, the methyl ether derivative of artemisinin, is widely used in the therapy of malaria [12]. Artemether also has potential therapeutic effects against various tumors by inhibiting tumoral proliferation and angiogenesis, as well as inducing apoptosis [13]–[16]. Furthermore, based on their liphophilicity, the derivatives of artemisinin including artemether tend to cross the blood-brain barrier, resulting in widespread distribution in brain tissues [17]–[19]. Therefore, the application of artemether in the therapy of gliomas has been paid more and more attention to by researchers. Nevertheless, the effects and mechanism of artemether in the therapy of malignant gliomas have been unclear so far by now.

Interactions between extracellular matrix (ECM) and adhesion molecules are essential for the development and angiogenesis of gliomas [20]. Glioma tissues express various adhesion molecules [21]. Vascular cell adhesion molecule-1 (VCAM-1) is one of the important cell surface adhesion molecules expressed by gliomas and its expression is positively correlated with the malignancy grades, suggesting that VCAM-1 expression is a relatively late phenomenon in tumorigenesis [21]. In addition, studies also showed that anti-VCAM-1 antibody could significantly inhibit the growth of the glioma and prolong the survival of tumor bearing rats [22]. Thus anti-VCAM-1 treatment may present a potential strategy for the therapy of gliomas. ECM is also considered to be a barrier against glioma metastasis. Matrix metalloproteinases (MMPs) are important proteolylic enzymes that degrade ECM, which is essential in the metastasis and invasion of gliomas. MMP-2 and MMP-9 contribute most in the malignancy of gliomas among other MMPs and are highly expressed in glioma tissues [23], [24]. In vitro study revealed that blocking of MMP-2 and MMP-9 resulted in inhibition of human malignant glioma cell invasion, indicating that MMP-2 and MMP-9 play an important role in the metastasis and invasion of glioma cells [25]. Phosphoinositide-3-kinase (PI3K) is an important signaling pathways linking extracellular signals to crucial cellular processes [26]. Studies have revealed that PI3K/Akt signaling pathway is activated in the invasion of gliomas and is involved in the regulation of VCAM-1 and MMP-2/9 expressions [27], [28].

In the present study, we first investigated the effects of artemether on the proliferation, migration, invasion and apoptosis of human glioma U87 cells. Based on this, RNA interference technique was employed to silence the expression of VCAM-1. Afterwards, we examined the effects of artemether combining with shRNA interference of VCAM-1 (shRNA-VCAM-1) on the cell migration, invasion, the expressions of MMP-2/9, phosphorylated Akt (p-Akt) and cell apoptosis, aiming at providing novel experimental evidence for the treatment of human brain glioma.

## Materials and Methods

### Cell Culture

The human U87 MG cell line was purchased from Nanjing KGI Biotechnology Co., Ltd. U87 MG cells were cultured in Dulbecco's Modified Eagle Medium: Nutrient Mixture F-12 (DMEM/F-12) (Hyclone, USA) supplemented with 10% fetal bovine serum (FBS) (Tianjin Hao Yang Biotech, China) in an incubator at 37°C in humidified atmosphere and 5% CO_2_.

### shRNA-VCAM-1 Stable Transfection

Cells were seeded into 24 well plates (Corning) until they reached 50–60% confluence prior to transfection. Afterwards, the stable transfection was performed. Four short hairpin RNAs (shRNA) targeting on VCAM-1 gene were designed and synthesized by Shanghai Jima pharmaceutical technology, China. The most applicable shRNA (shRNA-VCAM-1) was identified by G418 concentration gradient screening and applied in the following experiments. The sequence of shRNA-VCAM-1 was Sense: 5′-CACCGGAAGCCGATCACAGTCAAGTTTCAAGAGAACTTGACTGTGATCGGCTTCCTTTTTTG-3′; Antisense: 5′-GATCCAAAAAAGGAAGCCGATCACAGTCAAGTTCTCTTGAAACTTGACTGTGATCGGCTTCC-3′. The sequence of sh negative control (shNC) was Sense: 5′-CACCGTTCTCCGAACGTGTCACGTCAAGAGATTACGTGACACGTTCGGAGAATTTTTTG-3′; Antisense: 5′-GATCCAAAAAATTCTCCGAACGTGTCACGTAATCTCTTGACGTGACACGTTCGGAGAAC-3′. U87 cells were transfected by Lipofectamine LTX and plus reagent (Invitrogen) according to the manufacturer’s manual. The medium containing transfection reagents was replaced with DMEM/F-12 supplemented with 10% FBS 18 h after transfection. The cells were collected 48 h after transfection, processed in the following experiments and prepared for protein extraction. The silence efficiency of VCAM-1 was tested by Western blot (described below).

### Cell Viability and Proliferation Assay

Cell viability and proliferation activity was checked with MTT assay as described previously [29]. U87 cells were seeded into 96-well plates (Corning) at a density of 5,000 cells per well in DMEM/F-12 and incubated for 12 h under standard conditions (37°C and 5% CO2). The medium was replaced with either blank serum free DMEM/F-12 or DMEM/F-12 containing artemether (Yunnan Kunming Pharmaceutical Factory, China) in various concentrations (0, 50, 100, 200, 400, 600, 800 and 1000 µmol/L). Total volume in each well was 200 µL. U87 cells were incubated in these solutions for 24 h, 48 h and 72 h. Then 20 µL of MTT (5 mg/ml) was added into each well. After additional incubation for 4 h, the solution in each well was replaced with dimethyl sulfoxide (DMSO) (Sigma, USA) to solubilize formazan, the metabolic product of MTT. The plates were kept on a shaking mixer for 10 min to guarantee formazan was fully solubilized and the optical density was recorded at 490 nm using a microplate luminometer. Results are expressed as means ± SD. Data was analyzed by one-way ANOVA with the post hoc Tukey’s test applied for paired comparisons (*P*<0.05).

### Scratch Wound Healing Assay

Scratch wound healing assay was adapted to evaluate the migration ability of U87 cells according to the previous protocols [30], [31]. Briefly, cells were seeded into 6-well plates at the density of 1.0×10^5^/well until they reached 80% confluence. The scratching wounds were created in the monolayer of confluent U87 cells with a pipette tip. The width of wounds was assessed to be the same at the beginning of the experiments. The wells were rinsed with PBS three times to remove floating cells and debris. To test the effects of artemether on the migration of U87 cells, parental U87 cells were seeded and serum-free DMEM/F12 with or without artemether (0, 150, 300, 600 µmol/L) was added. Then these cells were incubated for 48 h. We also compared the effects of artemether with shRNA-VCAM-1. Parental U87 cells and shRNA-VCAM-1-U87 cells were seeded and either blank serum-free DMEM/F12 or serum-free DMEM/F12 containing artemether (300 µmol/L) was added in each cell line. The culture plates were incubated at 37°C and in 5% CO_2_. Wound healing was measured and recorded photographically over time using phase- contrast microscopy at 0, 24 and 48 h.

### In vitro Invasion Assay

The effects of artemether and shRNA-VCAM-1 on the invasion were checked using Transwell invasion assay with inserts of 8-µm pore size (Corning Costar) as described previously [32], [33]. The membranes of Transwell filter inserts were coated with Matrigel (BD Biosciences, USA) diluted with medium at the ratio of 1∶7. Parental U87 cells or shRNA-VCAM-1-U87 cells were trypsinized and resuspended in serum-free DMEM/F12 at the density of 3×10^4^/ml and 200 µl of U87 cell suspension was added into the upper chambers. 800 µl of DMEM/F12 supplemented with 10% FBS was placed in the lower chambers. Serum free DMEM/F12 served as a negative control. Artemether was added in the suspension of parental U87 cells at various concentrations (0, 150, 300, 600 µmol/L) and artemether at the concentration of 300 µmol/L was also added into the suspension of shRNA-VCAM-1-U87 cells. After incubated for 48 h, the inserts were taken out and cells remained on the upper surface of the filters were removed carefully with a cotton wool swab. The cells migrating to the underside surface were washed with PBS gently and fixed with methanol and glacial acetic acid (mixed at 3∶1) for 30 min at room temperature and stained in Giemsa stain for 15 min. The average number of invasive cells was counted in six random high-power fields (×400).

### Western Blot

U87 cells were stimulated as previously described. The cells were washed three times with ice-cold PBS to stop the stimulation. Then, the cells were collected and lysed in ice-cold radio immunoprecipitation assay lysis buffer containing 50 mM Tris (PH 7.4), 150 mM NaCl, 1% Triton X-100, 1% sodium deoxycholate, 0.1% SDS, sodium orthovanadate, sodium fluoride and EDTA (Beyotime Biotecnology, China) for 30 min. Then the pellet was disrupted with an ultrasonic crusher and samples were centrifuged at 17,000 rpm for 60 min at 4°C. The supernatant was collected as the soluble fraction and transferred to a new tube. The protein concentration of the soluble material was determined with BCA protein assay kit (Beyotime Biotecnology, China), with bovine serum albumin used as a standard. Equal amounts of proteins (20–25 µg) were separated by 12% SDS-polyacrylamide gel electrophoresis (PAGE) and processed for immunoblotting with a rabbit multiclonal antibody for VCAM-1 (diluted at 1∶100; Santa Cruz), a goat multiple clonal antibody for Akt1/2, a rabbit multiple antibody for p-Akt 1/2/3 (Ser473) and rabbit multiple clonal antibodies for MMP-2/9. A mouse polyclonal anti-β-actin antibody (diluted at 1∶2000; Zhongshan Goldenbridge Biotechnology, China) was used as an internal control. All the protein bands were scanned using ChemiImager 5500 V2.03 software, and the integrated density values (IDV) were calculated by computerized image analysis system (Fluor Chen 2.0) and normalized with that of β-actin.

### Kinetic Analysis of MMP-2/9 Activity

Kinetic analyses were performed to check the enzymatic activity of MMP-2/9 with fluorescently quenched gelatin as described previously [34]. OmniMMP fluorogenic substrate (Enzo Life) was prepared with DMSO at 4 µmol/L. The seqences of fluorescent substrates for MMP-2 and MMP-9 were Mca-Pro-Leu-Ala-Nva-Dap(Dnp)-Ala-Arg-NH_2_ and Mca-Arg-Pro-Lys-Pro-Val-Glu-Nva-Trp-Arg-Lys(Dnp)-NH_2_ seperately. The hydrolysis of these fluorescent substrates by protein supernatant (containing 20 nmol/L MMP-2/9, diluted with 50 mM HEPES, 10 mM CaCl_2_, 0.05% Brij-35, 10 µM ZnCl2 at pH 7.0) exacted from parental U87 cells or U87 cells treated with artemether (0, 150, 300, 600 µmol/L), shRNA-VCAM-1 and artemether (300 µmol/L)+shRNA-VCAM-1 was measured at 37°C. The rate of product formation in each well was recorded at 420 nm using a microplate luminometer and plot data was analyzed according to the manufacturer’s instruction. The activity of the MMPs was expressed as picomoles substrate hydrolyzed per second.

### Apoptosis Assay

Apoptosis of U87 cells was analyzed with Annexin V-fluorescein isothiocyanate (FITC)/Propidium iodide (PI) apoptosis detection kit (Keygen biotech, China). Briefly, cells were cultured in 6-cm dishes and treated with or without artemether (300 µmol/L) for 48 h. Then they were trypsinized with 0.25% Typsin, washed with PBS twice and collected by centrifugation (1,000 rpm, 5 min). The collection of cells was suspended with binding buffer at the density of 1×10^6^/ml, stained with Annexin V-FITC and PI in darkness for 15 min at room temperature and then analyzed by flow cytometry (BD Biosciences, USA).

### Statistical Analysis

All results were described as mean ± S.D. Statistical analysis was performed with SPSS 13 software. Differences between groups were assessed using a Student’s *t*-test and differences between multiple groups were assessed with Bonferroni test. *P*<0.05 was considered to be statistically significant.

## Results

### Artemether Inhibited the Proliferation of U87 Cells

**Figure 1:**
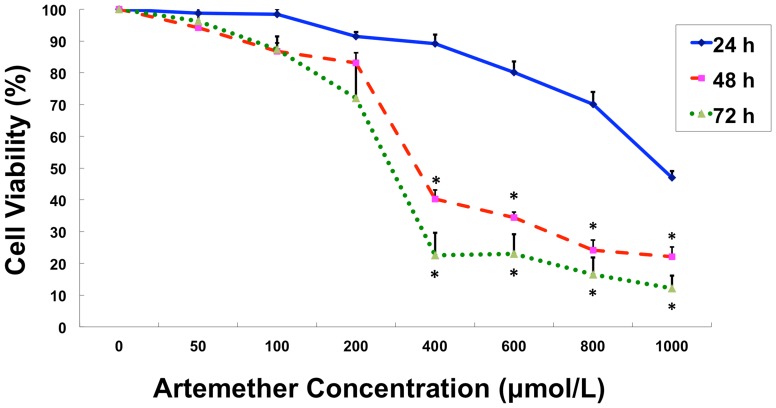
The effects of Artemether on the proliferation of U87 cells. Artemether inhibited the proliferation of U87 cells in time- and dose-dependent manners. U87 cells were treated with artemether at various concentration levels (0, 50, 100, 200, 400, 600, 800 and1000 µmol/L) for 24–72 h. 48-hour and 72-hour groups showed significant inhibitory effects on proliferation compared with the 24-hour group. Artemether inhibited the proliferation of U87 cells significantly at 400 µmol/L for 48 h or 72 h. Nevertheless, no significant difference was seen between 48-hour and 72-hour groups. n = 3. *P<0.01 (compared with 0 µmol/L group and 24-hour group).

The effects of artemether on the proliferation of U87 cells were assessed with MTT method. U87 cells were treated with artemether at various concentration levels (0, 50, 100, 200, 400, 600, 800 and1000 µmol/L) for 24–72 h. As shown in Figure 1, the results revealed that artemether inhibited the proliferation of U87 cells in time- and dose-dependent patterns. No obvious inhibitory effects on proliferation were obtained in 0–200 µmol/L groups. The cell viability was decreased significantly when treated at the concentration of 400 µmol/L (*P*<0.01). Moreover, significant proliferation inhibition was observed in 48-hour and 72-hour groups compared with the 24-hour groups.

Nevertheless, the cell viability did not vary significantly in different concentration levels above 400 µmol/L (600, 800 and 1000 µmol/L), nor did it change obviously in 48 or 72 h groups. These results indicated that artemether inhibited the proliferation of U87 cells significantly at 400 µmol/L for 48 h. Therefore 48 h was selected as the optimal administration time in the following studies. The IC50 for 24 h, 48 h and 72 h was 948.47±10.2 µmol/L, 354.81±13.8 µmol/L and 275.42±9.5 µmol/L respectively. Based on IC50 measured in this work, concentrations of 150, 300 and 600 µmol/L were selected to be used in the following experiments according to the literature [35]–[37].

### Artemether Inhibited the Migration and Invasion of U87 Cells

**Figure 2:**
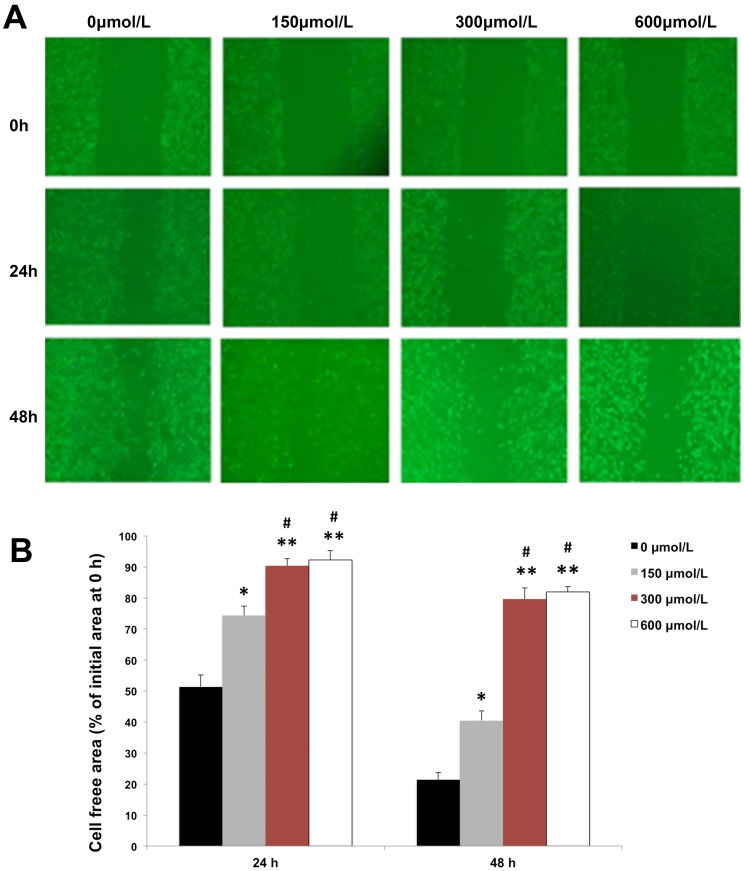
Artemether inhibited the migration of U87 cells. The effects of artemether on the migration of U87 cells were assessed by wound healing assay. (A) U87 cells were treated with artemether at various concentrations of 0, 150, 300 and 600 µmol/L for 48 h. (B) Statistical analysis of migrating cell numbers. Migration of U87 cells was inhibited with the increased concentration of artemether. The healing over scratch was inhibited significantly after 24 and 48 h from 150 µmol/L (*P*<0.05). The concentrations of 300 and 600 µmol/L displayed greater inhibitory effects (*P*<0.01). However, there was no significant difference between 300 and 600 µmol/L groups. (X200). n = 4. *P<0.05, **P<0.01 (compared with 0 µmol/L group), ^#^ *P*<0.05 (compared with 150 µmol/L group).

The effects of artemether on the migration and invasiveness of U87 cells were checked by the means of wound healing and Transwell assays. U87 cells were treated with artemether at various concentrations of 0, 150, 300 and 600 µmol/L for 0, 24 and 48 h. The results of wound healing assay displayed that healing over the scratch decreased gradually with increased concentrations of artemether (Figure 2). Artemether of 150 µmol/L displayed significant inhibition on the proliferation of U87 cell compared with 0 µmol/L group. More significant inhibition upon healing was observed at 300 µmol/L while the concentration of 600 µmol/L displayed the most significant inhibitory effect, indicating that artemether inhibited the migration of U87 cells in a dose-dependent manner.

**Figure 3:**
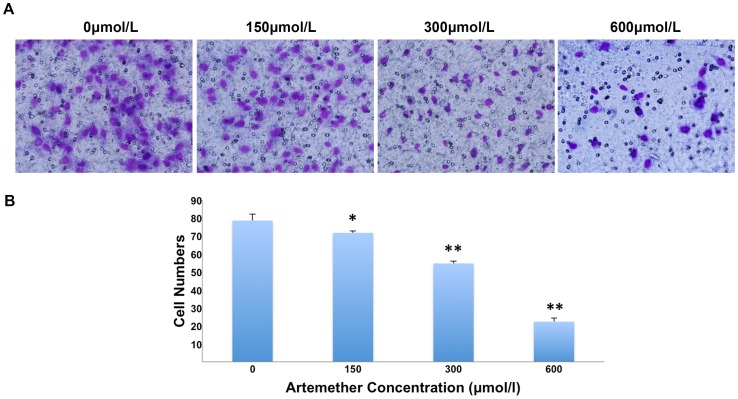
Artemether inhibited the invasiveness of U87 cells. (A) U87 cells were treated with artemether at various concentrations of 0, 150, 300 and 600 µmol/L for 48 h. Serum free L-DMEM served as a negative control. Cells migrating to the underside surface of Transwell filter were stained with Giemsa staining and photomicrographed, representing the invasiveness ability of U87 cells. (B) Statistical analysis of invasive cell numbers. The invasiveness of U87 cells was attenuated with the increased concentration of artemether. *n* = 4. *P<0.05, **P<0.01 (compared with 0 µmol/L group).

Then the effects of artemether on the invasiveness of U87 cells were assessed with Transwell assay. The results were shown in Figure 3, the invasiveness of U87 cells was significantly attenuated when treated with the concentrations of 150, 300 and 600 µmol/L (*P*<0.05, *P*<0.01, *P*<0.01 respectively).

### Artemether Inhibited the Expression and Activity of MMP-2 and MMP-9 on U87 Cells

**Figure 4:**
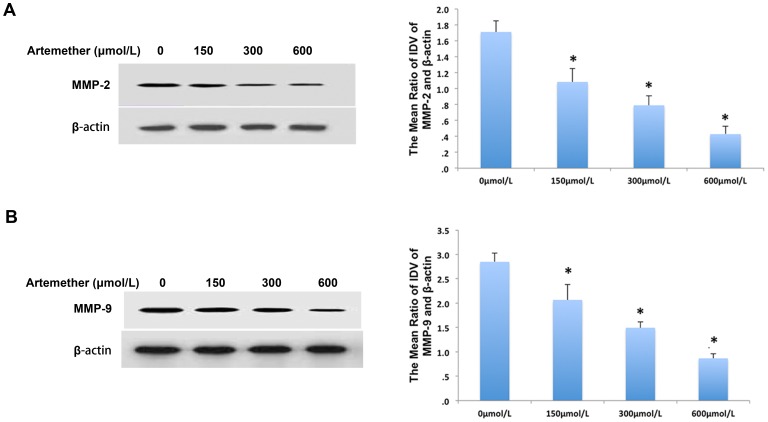
Artemether down-regulated the expression of MMP-2 and MMP-9 in U87 cells. U87 cells were treated with atemether at various concentrations (0 µmol/L, 150 µmol/L, 300 µmol/L and 600 µmol/L) for 48 h and Western Blot assay was utilized to check the changes of MMP protein expressions. (A) The effects of atemether on MMP-2 expression in U87 cells. (B) The effects of atemether on the expression of MMP-9. The concentrations of 150 µmol/L, 300 µmol/L and 600 µmol/L significantly inhibited the expression of MMP-2 and MMP-9 proteins compared with 0 µmol/L (*P<0.01). *n* = 4.

**Figure 5:**
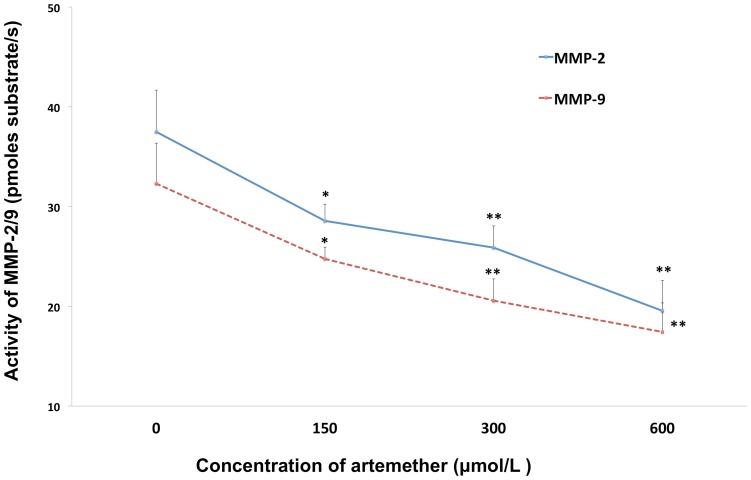
The effects of artemether on the enzymatic activity of MMP-2/9. Enzymatic activity of MMP-2/9 under the treatments of artemether (0, 150, 300, 600 µmol/L) was investigated by fluorescently quenched gelatin assay. The activity of the MMPs was expressed as picomoles substrate/s. The activity of both MMPs was inhibited significantly by artemether from the concentration of 150 µmol/L. *P<0.05; **P<0.01 (compared with control group). *n* = 4.

The family of MMPs has been identified as the invasion related proteins. MMP-2 and MMP-9 are two important members, which play important roles in the invasion and malignancy of glioma cells [23]. To investigate whether artemether could regulate the expression of MMP-2 and MMP-9, U87 cells were treated with artemether at various concentrations for 48 h and Western Blot assay was utilized to check the changes of MMP protein expressions. The concentrations of 150 µmol/L, 300 µmol/L and 600 µmol/L significant inhibited the expression of MMP-2 (Figure 4A) and MMP-9 (Figure 4B) compared with 0 µmol/L (*P*<0.01). 600 µmol/L displayed the maximum inhibition on both protein expressions. The kinetic analysis of catalyzed hydrolysis using a synthetic fluorogenic substrate was performed to investigate the effects of artemether on the catalytic behavior of MMP-2 and MMP-9 according to the literature [34]. Results showed that the enzymatic activity was reduced significantly with the treatments of arthemeter in a dose-dependent manner (Figure 5).

### Artemether Combined with shRNA-VCAM-1 Displayed Synergistic Inhibitory Effects on the Migration, Invasion of U87 Cells

**Figure 6:**
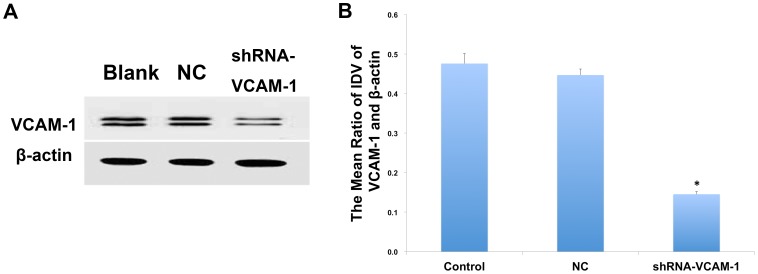
The silence efficiency of VCAM-1 in U87 cells. The expression of VCAM-1 protein was checked with Western Blot. (A) The results of Western Blot. (B) Statistical analysis. The expression of VCAM-1 protein was inhibited significantly in the shRNA-VCAM-1 group compared with blank group. NC control group showed no obvious difference with blank group. The silence efficiency of VCAM-1 was 70%. *P<0.01 (compared with blank group). *n* = 4.

It has been reported that VCAM-1 is highly expressed in the glioma tissues. VCAM-1 mediates the migration of leukocytes and messenchymal stromal cells. Nevertheless, the role of VCAM-1 in the migration and invasion of glioma cells has not been clearly identified. In the present study, we also tried to investigate the role of VCAM-1 in the malignancy of U87 cells and whether the inhibitory effects would be enhanced if combined with shRNA-VCAM-1. The expression of VCAM-1 was inhibited with shRNA stable transfection technology. The silence of VCAM-1 protein was assessed with Western Blot and the results were displayed in Figure 6. The expression of VCAM-1 was inhibited significantly in the shRNA-VCAM-1 group compared with blank group. NC control group showed no obvious difference with blank group. The silence efficiency of VCAM-1 was 70%.

**Figure 7:**
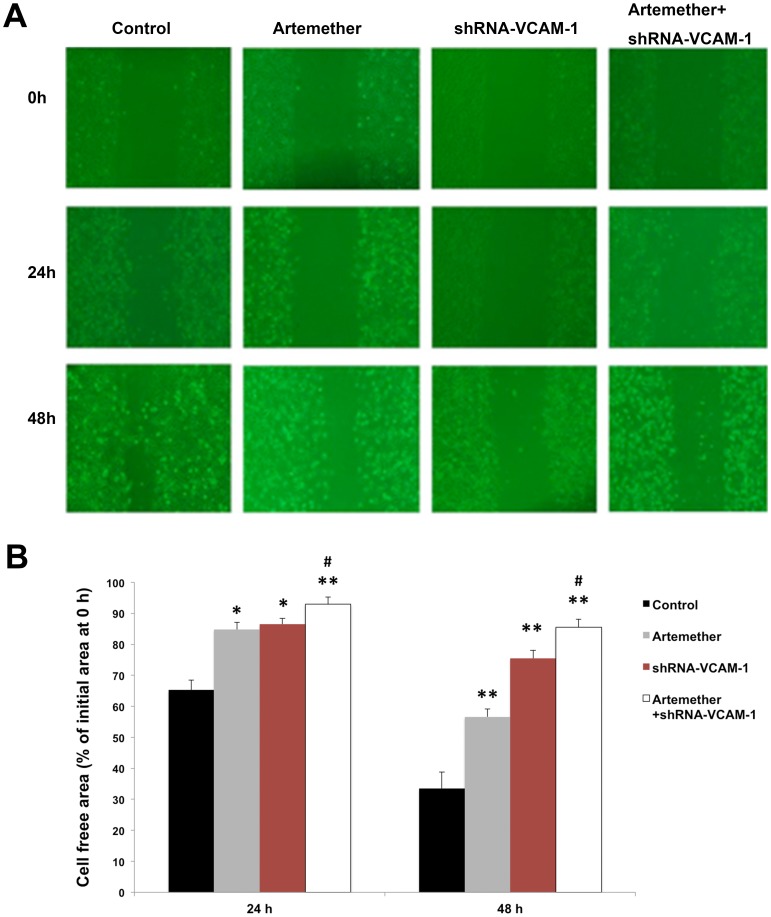
The effects of shRNA-VCAM-1 alone or combined with artemether upon the migration capacity of U87 cells. The effects of artemether on the migration of U87 cells were assessed by wound healing assay. (A) Parental U87 cells and shRNA-VCAM-1-U87 cells were treated with or without artemether (300 µmol/L, 48 h). (B) Statistical analysis of migrating cell numbers. The migration of shRNA-VCAM-1 U87 cells was inhibited significantly compared with control group and the artemether+shRNA-VCAM-1 group displayed the greatest inhibitory effects. (X200). *P<0.05, **P<0.01 (compared with the control group); ^#^ *P*<0.05, (compared with artemether and shRNA-VCAM-1 groups). *n* = 4.

**Figure 8:**
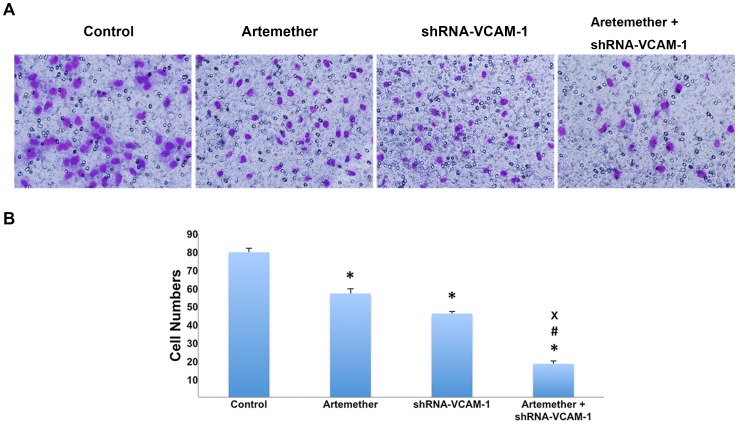
The effects of shRNA-VCAM-1 alone or combined with artemether on the invasiveness of U87 cells. (A) U87 cells were treated with artemether of 300 µmol/L, shRNA transfected or treated with both. Serum free L-DMEM served as a negative control. Cells migrating to the underside surface of Transwell filter were stained with Giemsa staining and photomicrographed, representing the invasiveness ability of U87 cells. (×400). (B) Statistical analysis of invasive cell numbers. The invasiveness of shRNA-VCAM-1 U87 cells was inhibited significantly compared with control group and the artemether+shRNA-VCAM-1 group displayed the maximum inhibitory effects. *n* = 4. *P<0.01 (compared with the control group); ^#^ *P*<0.01, (compared with artemether group); ^×^ *P*<0.01 (compared with shRNA-VCAM-1 group).

Next, the role of VCAM-1 in the migration and invasiveness of human glioma cells was investigated with shRNA-VCAM-1 U87 cells. The results of wound healing assay revealed that migration of shRNA-VCAM-1 U87 cells was inhibited significantly compared with control group (Figure 7). Similar results were observed in Transwell assays, the invasiveness of U87 cells was inhibited with the silence of VCAM-1 (Figure 8). To investigate the enhancing effects of artemether combined with shRNA-VCAM-1, the shRNA-VCAM-1 U87 cells were treated with artemether (300 µmol/L). Interestingly, the artemether+shRNA-VCAM-1 group displayed even greater inhibitory effects than shRNA-VCAM-1 or artemether group in both migration and invasiveness assays (Figure 7, 8).

### Artemether Combined with shRNA-VCAM-1 Synergistically Inhibited the Expression and Activity of MMP-2 and MMP-9 on U87 Cells

**Figure 9:**
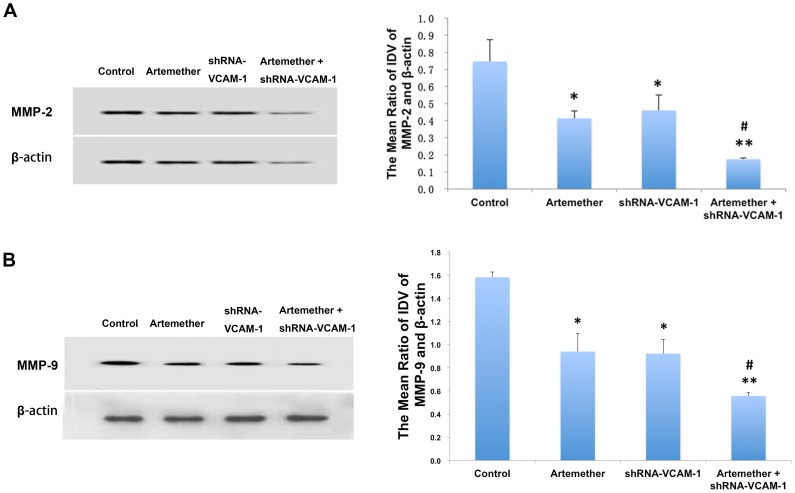
The effects of artemether, shRNA-VCAM-1 and artemether combined with shRNA-VCAM-1 on the expression of MMP-2 and MMP-9. U87 cells were treated with artemether of 300 µmol/L, shRNA transfected or treated with both. Serum free L-DMEM served as a negative control. Expressions of MMP-2/9 were checked with Western Blot. (A) Effects of artemether, shRNA-VCAM-1 and artemether combined with shRNA-VCAM-1 on the expression of MMP-2. (B) The effects of artemether, shRNA-VCAM-1 and artemether combined with shRNA-VCAM-1 on the expression of MMP-9. Statistical analysis showed that artemether and shRNA-VCAM-1 significantly reduced the expression levels of both MMP-2 and MMP-9 proteins compared with control group. Artemether+shRNA-VCAM-1 displayed the maximum inhibition on the expression of MMPs compared with single artemether or shRNA-VCAM-1 groups. *P<0.05, **P<0.01 (compared with control group); ^#^ *P*<0.05 (compared with artemether or shRNA-VCAM-1 group). *n* = 4.

**Figure 10:**
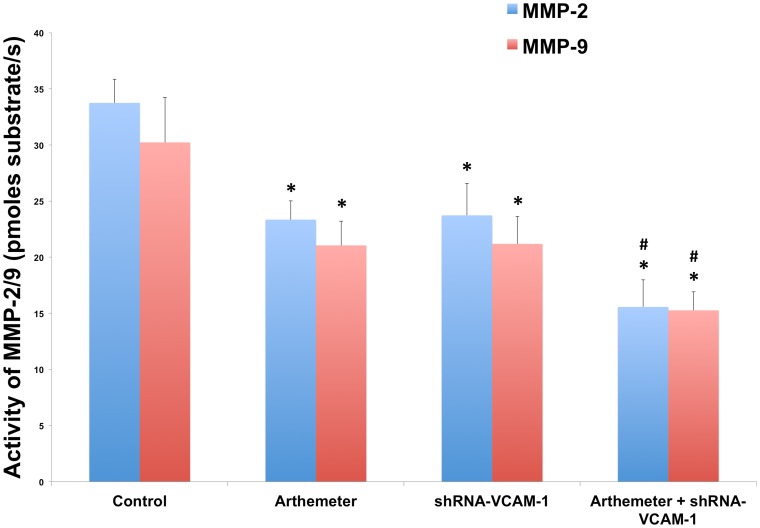
The effects of artemether, shRNA-VCAM-1 and artemether combined with shRNA-VCAM-1 on the enzymatic activity of MMP-2/9. Enzymatic activity of MMP-2/9 under the treatments of artemether (300 µmol/L), shRNA-VCAM-1 and artemether combined with shRNA-VCAM-1 was investigated by fluorescently quenched gelatin assay. The activity of the MMPs was expressed as picomoles substrate/s. The activity of both MMPs was inhibited significantly by artemether and shRNA-VCAM-1 compared with the control group. Artemether+shRNA-VCAM-1 displayed even greater inhibitory effect on enzymatic activity over single artemether or shRNA-VCAM-1 treatments. *P<0.05 (compared with control group); ^#^ *P*<0.05 (compared with artemether or shRNA-VCAM-1 group). *n* = 4.

In order to investigate the effects of shRNA-VCAM-1 and artemether combining with shRNA-VCAM-1 on the expressions of MMP-2 and MMP-9, Western Blot assay was used in control, shRNA-VCAM-1 and artemether+shRNA-VCAM-1 groups. As described above, data revealed that artemether inhibited the expressions of MMP-2 and MMP-9 in U87 cells (Figure 4). Similarly, shRNA-VCAM-1 significantly reduced the expression levels of both MMP-2 (Figure 9A) and MMP-9 (Figure 9B) (*P*<0.01). Moreover, artemether+shRNA-VCAM-1 displayed the greatest inhibition on the expression of MMPs over single artemether or shRNA-VCAM-1 groups (*P*<0.05). The enzymatic activity was reduced significantly with the treatments of arthemeter or shRNA-VCAM-1 compared with control group and artemether+shRNA-VCAM-1 group displayed the maximum inhibition on the MMP activity (Figure 10).

### Artemether Combined with shRNA-VCAM-1 Promoted the Apoptosis of U87 Cells

**Figure 11:**
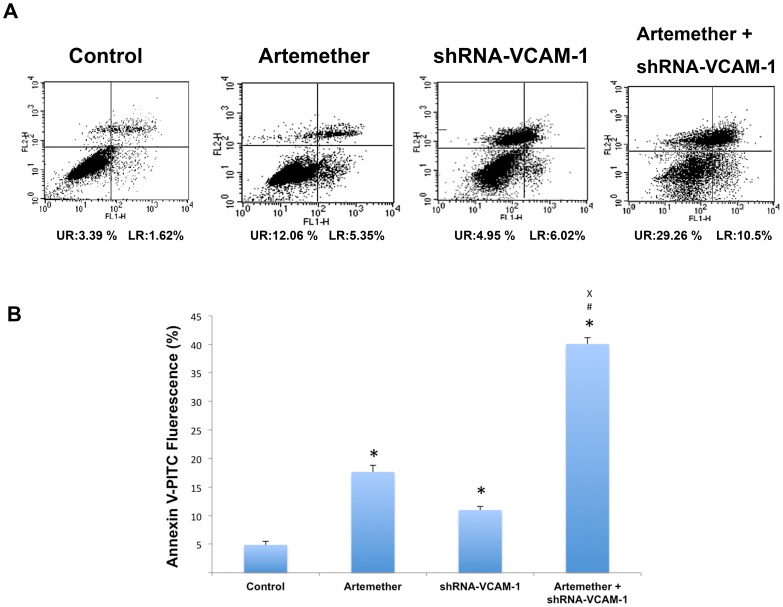
The effects of artemether, shRNA-VCAM-1 and artemether combined with shRNA-VCAM-1 on the apoptosis of U87 cells. (A) U87 cells were treated with artemether (300 µmol/L) and shRNA-VCAM-1 alone or in combination. The apoptosis was evaluated by Annexin V-FITC/PI staining. (B) Statistical analysis. Artemether and shRNA-VCAM-1 significantly promoted the apoptosis of U87 cells compared with the control group and artemether+shRNA-VCAM-1 displayed the maximum promoting effect. *n* = 4. *P<0.01 (compared with control group); ^#^ *P*<0.01 (compared with artemether group); ^×^ *P*<0.01 (compared with shRNA-VCAM-1 group).

The effects of artemether (300 µmol/L) and shRNA-VCAM-1 alone or in combination on the apoptosis of U87 cells were also investigated. The apoptosis was evaluated by Annexin V-FITC/PI staining. The results were shown in Figure 11. The rates of apoptosis in control group, artemether group, shRNA-VCAM-1 group and artemether+shRNA-VCAM-1 group were 4.89±0.58%, 17.66±1.12%, 10.97±0.62% and 40.04±1.11% respectively. As shown in Figure 11B, artemether and shRNA-VCAM-1 significantly promoted the apoptosis of U87 cells compared with the control group (*P*<0.01) and artemether+shRNA-VCAM-1 displayed the maximum promoting effect on the apoptosis over artemether or shRNA-VCAM-1 alone (*P*<0.01).

### Artemether Combined with shRNA-VCAM-1 Synergistically Inhibited the Expression of p-Akt

**Figure 12:**
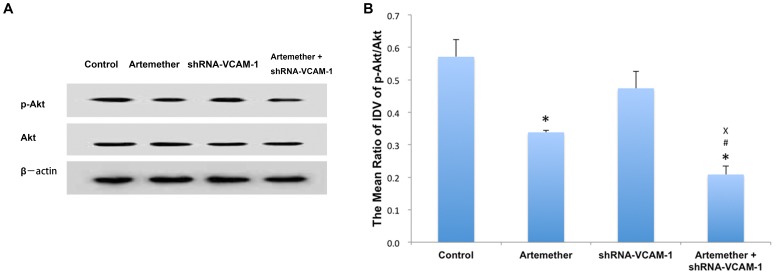
The effects of artemether, shRNA-VCAM-1 and artemether combined with shRNA-VCAM-1 on the expression of p-Akt. (A) U87 cells were treated with artemether (300 µmol/L) and shRNA-VCAM-1 alone or in combination. (B) Statistical analysis. The expression of p-Akt was checked with Western Blot. p-Akt was significantly inhibited in artemether and artemether+shRNA-VCAM-1 groups compared with the control group. Artemether+shRNA-VCAM-1 produced further inhibition on p-Akt expression. *n* = 4. *P<0.01 (compared with control group); ^#^ *P*<0.05 (compared with artemether group); ^×^ *P*<0.01 (compared with shRNA-VCAM-1 group).

Phosphoinositide-3-kinase (PI3K)/Akt pathway is involved in many biotic activities. It has also been clarified that PI3K/Akt pathway also plays a major role in the malignancy of glioma cells such proliferation and invasiveness [27]. In the present study, we also investigated the alteration of phosphorylated Akt (p-Akt) under the pre-treatments of artemether, shRNA-VCAM-1 and artemether+shRNA-VCAM-1 (as described above). The results of Western Blot were shown in Figure 12. Compared with the control group, the expressions of p-Akt were significantly inhibited in artemether and artemether+shRNA-VCAM-1 groups (*P*<0.01). However, no significant inhibition was obtained in the shRNA-VCAM-1 group (*P*>0.05). Interestingly, artemether+shRNA-VCAM-1 produced further inhibition on p-Akt expression than artemether alone (*P*<0.01).

## Discussion

In the present study, we discovered that artemether inhibited the proliferation, migration and invasion of human malignant glioma U87 cell line in a dose-dependent manner. We also showed that artemether significantly promoted the apoptosis of U87 cells and inhibited the expressions of MMP-2 and MMP-9, which are the two major proteolylic enzymes involved in the invasion and metastasis of gliomas [23], [24]. Based on these results, we further investigated the role of VCAM-1 in the malignant behavior of glioma cells with RNA interfering silence technology. The shRNA interference of VCAM-1 (shRNA-VCAM-1) also resulted in the inhibition of migration, invasion as well as the expression of MMP-2 and MMP-9 in U87 cell line and also significantly promoted the apoptosis. Interestingly, the treatment of artemether combined with shRNA-VCAM-1 displayed greater inhibitory effects on the malignancy of U87 cells. To our knowledge, this is the first study revealing the effects of artemether and VCAM-1 knockdown on the malignant behavior of glioma cells.

Artemisinin is the product isolated from the plant of Artemisia annua, a Chinese traditional herb. Artemisinin and its derivatives are originally used in the therapy of malaria, especially antidrug malaria [8], [12], [38], [39]. In recent years, studies have revealed that artemether, the methyl ether derivative of artemisinin also has potential therapeutic effects against various tumors by inhibiting tumoral proliferation, angiogenesis as well as inducing apoptosis [13]–[16]. Alcântara et al found that artemether promoted the apoptosis and necrosis of human gastric cancer cell PG1100 by inducing DNA damage [14]. Hou et al reported that artemether displayed cytotoxicity in human hepatoma cells [40]. However, there are very few studies about the role and mechanism of artemether in the therapy for brain tumors, especially for gliomas. Wu et al reported that artemether decreased the microvessel density and volume of C6 gliomas subcutaneous planted in SD rats, indicating that artemether inhibited the glioma growth and angiogenesis [15]. The inhibitory effects of artemether was also observed in the therapy of pituitary adenoma [41]. In vitro study revealed that artemether inhibited the proliferation of mouse Nb2a neuroblastoma cells and rat C6 glioma cells significantly at the concentration of 100 µmol/L [42]. We investigated the inhibitory effects on human U87 glioma cells from 50 µmol/L (0, 50, 100, 200, 400, 600, 800 and 1000 µmol/L). Results indicated that the proliferation of U87 cells was inhibited significantly at 400 µmol/L, while there was no obvious difference in 0, 50, 100 and 200 µmol/L groups. The IC50 was 354.81±13.8 µmol/L at 48 h in this study, which was different from the literature. Efferth et al studied the effects of artemether on 55 tumor cell lines and the IC50 value was 82.4 µmol/L [16]. The difference may be due to the followings reasons: first, the cell lines differed and the sensitivities to artemether might be different; second, detecting assays were different. IC50 represents a measure of the effectiveness of an agent in inhibiting biological or biochemical function and inhibitory study of antitumor agents should be performed at a concentration based on IC50 [35]–[37]. Therefore concentrations of artemether used in the following experiments were set at 150, 300 and 600 µmol/L. Artemether inhibited the U87 cell migration and invasion capacity and promoted the apoptosis of U87 cells at the concentration of 300 µmol/L while the apoptosis rate was up to 15%. Our results were consistent with the previous studies, showing that artemether might be a potential therapeutic agent for gliomas.

As an important adhesion molecule, VCAM-1 plays an important role in the migration and proliferation of endothelial cells to induce angiogenesis [43]. VCAM-1 was also related to the pathological process of the tumors. For example, Ding et al found that the expression of VCAM-1 correlated with the microvascular density of gastric cancer, which may be involved in the process of angiogenesis and metastasis of gastric cancer [44]. Maurer et al indicated that the interaction of VCAM-1 and its ligand (VLA-4) was the key point in the tumor metastasis [45]. Glioma tissues express higher level of VCAM-1 compared with normal brain tissue [21]. VCAM-1 is also the key factor in the glioma induced bone marrow stromal stem cell (BMSCs) migration [28]. The anti-VCAM-1 treatment may be a new potential therapeutic strategy for malignant tumors. RNA interference silence of VCAM-1 inhibited melanoma migration and invasion [46]. Chemotherapeutic agents such as cisplatin inhibit the migration and invasion of small cell lung cancer through decreasing the expression of VCAM-1 [47]. The application of anti-VCAM-1 antibody significantly inhibited the growth of the glioma transplanted into rats and prolong the survival of tumor bearing rats [22]. In this study, we also aimed to investigate the effects of VCAM-1 in the malignance of U87 cells by the mean of RNA interference silence. Results demonstrated that shRNA-VCAM-1 significantly inhibited the migration and invasion of U87 cells and promoted the apoptosis. Artemether combined with shRNA-VCAM-1 displayed greater synergistic inhibitory effects against U87 glioma cells than the treatment of artemether or shRNA-VCAM-1 alone. These results have provided new theoretical basis for the treatments of gliomas.

**Figure 13:**
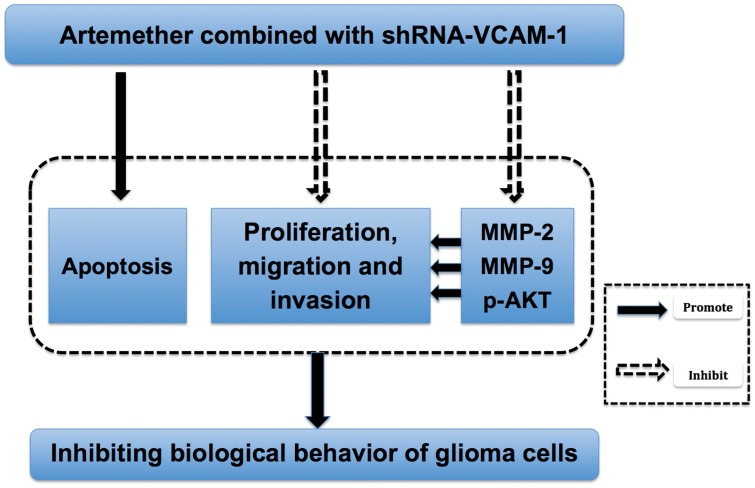
The schema of the effects of artemether and shRNA-VCAM-1 on the U87 human glioma cells. The schematic diagram demonstrates the effects of artemether and shRNA-VCAM-1 on the malignant behavior of U87 human glioma cells. The combination of artemether with shRNA-VCAM-1 shows a synergistic inhibitory effect on the malignancy of glioma cells by inhibiting the migration, invasion of glioma cells and promoting the apoptosis of U87 human glioma cells through inhibiting MMP-2, MMP-9 and p-Akt expressions.

The mechanisms relating the inhibitory effects of artemether and shRNA-VCAM-1 on the malignancy of glioma cells remain poorly understood. Previous study has revealed that VCAM-1 was involved in the regulation of MMP-2 and MMP-9 [48]. The two MMPs are the most important proteolylic enzymes that degrade extracellular matrix to provide efficient space for glioma to extend, which is essential in the metastasis and invasion of gliomas [23]–[25]. Similarly, Our results revealed that artemether and shRNA-VCAM-1 inhibited MMP-2 and MMP-9 expressions and activity on U87 cells. In addition, the combined treatment of artemether with shVCAM-1 showed a synergistic effect on inhibiting the migration and invasion of U87 cells. The activation of PI3K/Akt signaling pathway may be involved in the biological behavior of many tumors. Increased PI3K/Akt signaling is also associated with increased invasiveness and MMP activity [27]. This signaling pathway was activated to reduce apoptosis in the presence of various malignant tumors and diseases [49]–[52]. The inhibition of PI3K and Akt resulted in inhibition of tumor growth/invasion and promotion of glioma apoptosis in vitro and in vivo [53]–[55]. In the present study, inhibition of p-Akt expression was also observed under the treatments of artemether and shRNA-VCAM-1 and the inhibitory effect was strengthened with the their combined administration. These results indicated that the inhibitory effects of artemether and shRNA-VCAM-1 might be associated with the inhibition of MMP-2, MMP-9 and p-Akt expressions. Combining the results in present study and previous literature, artemether combined with shRNA-VCAM-1 showed a synergistic inhibitory effect on angiogenesis, proliferation, migration and invasion of glioma cell as well as promotion of apoptosis through inhibition of MMP-2, MMP-9 and PI3K/Akt pathway, resulting in the decline of malignancy of glioma cells (summarized in Figure 13). Nevertheless, the applications of artemether and anti-VCAM-1 in glioma therapy and detailed molecular mechanisms merit further studies.

### Conclusions

Artemether reduces the migration and invasion capability of glioma cells and promotes the apoptosis of U87 human glioma cells by inhibiting MMP-2, MMP-9 and p-Akt expressions. The combination of artemether with shRNA-VCAM-1 shows a synergistic inhibitory effect on the malignancy of glioma cells.
